# Focus on perovskite emitters in blue light-emitting diodes

**DOI:** 10.1038/s41377-023-01206-2

**Published:** 2023-07-24

**Authors:** Xiaoyu Yang, Li Ma, Maotao Yu, Hao-Hsin Chen, Yongqiang Ji, An Hu, Qixuan Zhong, Xiaohan Jia, Yanju Wang, Yuzhuo Zhang, Rui Zhu, Xinqiang Wang, Changjun Lu

**Affiliations:** 1 Leyard Optoelectronic Co., Ltd, 100091 Beijing, China; 2 State Key Laboratory for Artificial Microstructure and Mesoscopic Physics, School of Physics, Frontiers Science Center for Nano-optoelectronics & Collaborative Innovation Center of Quantum Matter, Peking University, 100871 Beijing, China; 3 State Key Laboratory of Chemical Resource Engineering, College of Materials Science and Engineering, Beijing University of Chemical Technology, 100029 Beijing, China; 4 Peking University Yangtze Delta Institute of Optoelectronics, 226010 Nantong, Jiangsu China

**Keywords:** Organic LEDs, Inorganic LEDs

## Abstract

Blue perovskite light-emitting diodes (PeLEDs) are essential in pixels of perovskite displays, while their progress lags far behind their red and green counterparts. Here, we focus on recent advances of blue PeLEDs and systematically review the noteworthy strategies, which are categorized into compositional engineering, dimensional control, and size confinement, on optimizing microstructures, energy landscapes, and charge behaviors of wide-bandgap perovskite emitters (bandgap >2.5 eV). Moreover, the stability of perovskite blue emitters and related devices is discussed. In the end, we propose a technical roadmap for the fabrication of state-of-the-art blue PeLEDs to chase and achieve comparable performance with the other two primary-color devices.

## Introduction

Next-generation displays tend to behave at low cost, ultrahigh-resolution, and wide color gamut, wherein the emerging perovskite light-emitting diode (PeLED) rises in good response to these features, arousing more research passion on three primary-color PeLED devices^1–6^. Rapid progress has been made in red and green devices in the current stage^7,8^, while the development of blue PeLEDs is lagging in all respects, especially their inferior device efficiencies^9,10^. In contrast, in traditional III–V-based LEDs, blue chips are the best-performing among the three colors; while the chips with long-wavelength emissions, especially the red with complex composition and manufacturing process, face serious indium doping and downsizing effects^11,12^. Fortunately, the color conversion could be adopted to avoid these weaknesses by applying blue chips to photo-excite the narrow-bandgap emitters, such as phosphor, II–VI quantum dots, or perovskites, for efficient long-wavelength (>550 nm) emissions^13,14^. However, for the perovskite-based displays, color conversion seems unworkable at present due to the poor performance of blue PeLEDs. In brief, further improving the blue perovskite emitters and resultant PeLED devices is the sole route to realize all-perovskite displays.

**Fig. 1:**
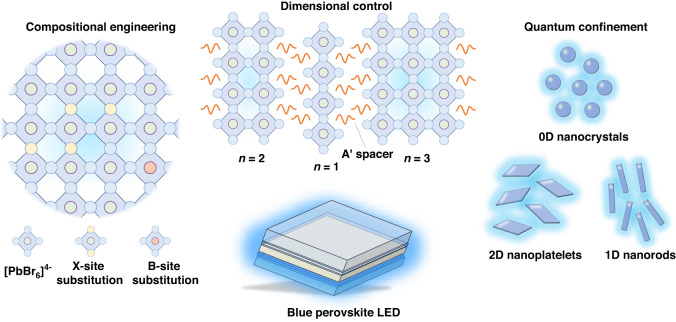
Strategies on bandgap enlargement for blue perovskite emitters. The structural formula of the metal-halide perovskite is ABX_3_, where A denotes monovalent cation, B denotes metal cations, and X generally denotes halide anions. *n* denotes the number of B-X octahedron layers between large intercalating cations

Thanks to the high tolerance and soft lattice of metal-halide (B-X) perovskites^15–17^, there is a wealth of strategies to enlarge the bandgap for blue emission (Fig. 1). The perovskite band structure is sensitive to the electron cloud distribution of the B-X octahedral scaffold^16^. X-site substitution, for example, from Br to Cl in CsPbX_3_, could significantly adjust the bandgap from 2.4 (green) to ≥3.0 eV (blue)^18^, which is the most common strategy for bandgap enlargement. Moreover, cations in perovskites indirectly influence band structures by the structural deformation of the B-X octahedron^19^. Introducing large cations to partially replace common small counterparts in perovskite precursors could promote the formation of low-dimensional phases. The in situ formed quantum wells by high-dielectric intercalating cations result in space confinement and energy cascade, collectively contributing to blueshift emissions. Space confinement is found in as-formed perovskite nanocrystals (NCs) as well^20^. The crystals with at least one dimension less than several nanometers, such as zero-dimensional (0D) quantum dots (QDs), 1D nanowires (NWs), and 2D nanoplatelets (NPLs), realize enlarged bandgaps and near-unity photoluminescence quantum yield (PLQY) in blue emission. However, the above strategies face various obstacles to fabricating high-performance blue PeLEDs. Cl-substitution generally introduces deep-level trap states as nonradiative recombination centers and aggravates device degradation because of Cl-involved phase segregation. Low-dimensional perovskites are difficult to be arranged into well-organized phase distribution with rational energy landscapes. NCs present outstanding PLQYs but cannot exploit their full potential when integrated into devices, which are limited by their surface-insulating ligands and unmatched band alignment.

Here, we focus on three classes of bandgap-opening strategies on blue perovskite emitters, which include compositional engineering, dimensional control, and size confinement. Representative advances are systematically reviewed and discussed to clarify the keys to the improvement of perovskites and PeLED devices from a bottom-up perspective. A technical roadmap is proposed for the state-of-the-art blue PeLED in both efficiency and stability views, establishing meaningful guidance for future perovskite-based displays.

## Compositional engineering

**Fig. 2:**
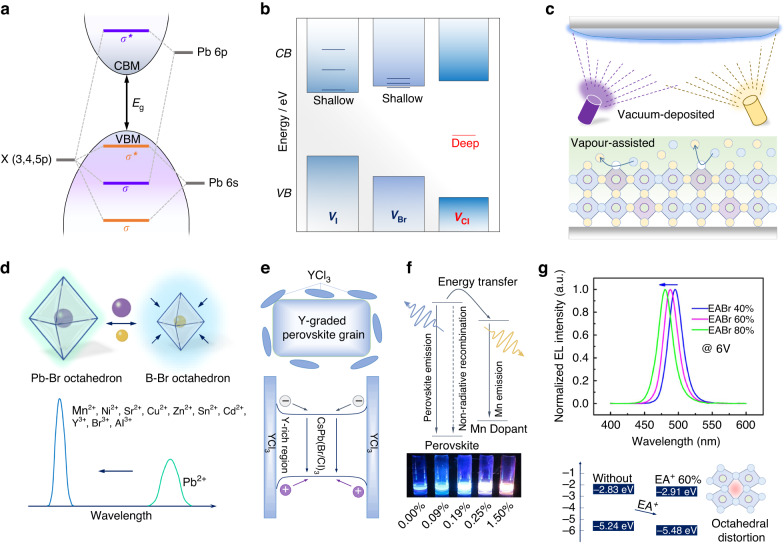
Compositional engineering. **a** The representative electronic structure of Pb-X perovskites. *σ* and σ^*^ denote the bonding and antibonding orbitals, respectively. *E*_g_ denotes the bandgap. **b** Relatively thermodynamic ionization levels of the halide vacancies (V_I_, V_Br_, V_Cl_) in MAPbI_3_, MAPbBr_3_, and CsPbCl_3_. **c** The vapor-assisted (bottom) and vacuum-deposited (top) methods for the fabrication of CsPb(Br,Cl)_3_ films. **d** Schematics of the contracted Pb-X octahedral lattices upon B-site substitution, and the enhanced blue-shifted PL emission by a variety of substitution candidates. **e** Energy diagram of the Mn-doped perovskite (top), and the PL picture of CsMn_*y*_Pb_1-*y*_Br_*x*_Cl_3-*x*_ with different doping content of Mn^2+^ (bottom). **f** The distribution and corresponding energy diagram of the Y-doped CsPb(Br,Cl)_3_ grain. **g** Normalized electroluminescence (EL) spectra of the blue PeLED devices under 6 V bias with the increment of EABr (top). The energy diagram of the CsPbBr_3_ with and without 60% EA^+^ substitution and the schematic of octahedral distortion (bottom). **e** is reprinted from ref. ^35^ with permission from Elsevier

The electronic structure of crystalline semiconductors is determined by the lattice type and composition^21^. The arrangement of energy bands is hybridized from atomic orbitals and can be adjusted by the atom substitution or applied lattice strain/stress^22^. The conventional electronic structures of the Pb-X perovskite are shown in Fig. 2a. The atomic orbitals of Pb 6 *s*, Pb 6*p*, and X *p* are involved in the construction of the band edge, where the Pb 6*p*-X *p* hybrid antibonding orbital contributes to the conduction band minimum (CBM) and the Pb 6*s*-X *p* bonding orbital dominants the valence band maximum (VBM), collectively determining the bandgap^16,19^. The bandgap will be changed with the overlapping degree of Pb-X orbitals and, therefore, the atom substitution with varied orbital overlaps is the most intuitive strategy to enlarge the bandgap for blue emission.

### X-site substitution

Conventional halide species in Pb-X perovskites are I, Br, and Cl, which satisfies the structural tolerance factor of the ABX_3_ perovskite lattice^23^. When I is replaced by smaller Br or Cl, there is a contraction of the Pb-X lattice, resulting in bandgap enlargement. In this case, for the 3D perovskite emitters with blue emissions, X-site is generally composed of mixed Br/Cl. While, except for the variation of energy bands, X-site substitution would unpredictably introduce additional trap states or non-ideal ionic behaviors. For example, halide vacancies (V_X_) are common perovskite defects because of their low formation energies^24^. From the simulation results (Fig. 2b), a deep-level state presents in the middle of the band at the existence of V_Cl_; while the energy levels of V_I_ and V_Br_ locate at the edge of conduction bands that identified as shallow defects^25–27^. That means in comparison to Pb–I or Pb–Br perovskites, Cl-involved counterparts will suffer from additional V_Cl_-induced nonradiative recombination paths. From Br to Br/Cl-mixed perovskites, there is a significant reduction of PLQYs despite the bandgap opening, which is one of the main factors for inferior emissions of blue perovskites than the other two colors.

Phase segregation is the weakness of halide-mixed perovskites^28^, where the Br/Cl-mixed perovskites are no exception^29^. It is more pronounced in high-Cl perovskites (i.e., Cl >30%) that are generally desired for pure-blue emissions. Therefore, the development of Br/Cl-mixed blue PeLED devices is impeded by poor color stabilities due to the Br/Cl segregation under applied bias. The halide heterogeneity in perovskite films, which derives from the solubility difference between halide species in precursor^30,31^, is critical for triggering the subsequent phase segregation. The challenge is to suppress the overly fast crystallization of Cl species, such as the CsCl with poor solubility in precursor, without foreign additives or solvent replacement. A vapor-assisted method aiming at the as-casted wet intermidiates^32^, which provides a favorable environment for efficient ion diffusion and rearrangement, wins great success in halide homogenization (Fig. 2c). The dimethylformamide atmosphere prolongs the liquid intermediate of the perovskite film, achieving a good balance of the chemical potentials between Br-dominated liquids and Cl-dominated solids during film formation to the final homogenized constituents. Moreover, vacuum co-evaporation is a good solution for film heterogeneities^33^. Without solubility limitation, Cl sources with Br sources are feasibly deposited with desired compositional proportion and homogeneous distribution. Based on this strategy, pure-blue emission of high-Cl perovskites is facile to implement by vacuum co-evaporation. Although original film heterogeneities and defects improved to a respectable level, the following halide segregation is still unavoidable because of intrinsic low activation energies of halides that could cause serious ion migration. As mentioned, the lattice contraction of the B-X octahedron is the reason for bandgap opening, and similarly, B-site substitution is another pathway.

### B-site substitution

Replacing Pb^2+^ with a smaller cation could contract the Pb–Br octahedron, as many divalent or trivalent candidates have been used to enlarge the bandgap (Fig. 2d)^34–40^. Almost all the substitutions are accompanied by improved radiative efficiencies of perovskite films. For example, smaller Ni^2+^ owns the superiority of the coordination with halides to form octahedrons^36^, avoiding the Ni-related impurities as potential recombination centers. According to the simulation, the introduction of Ni^2+^ effectively increases the formation energies of V_Cl_ and the total system, contributing to a smaller resultant perovskite domains and significantly enhanced PLQYs. Interestingly, Sn^2+^, generally adopted as the bandgap-narrowing agent, is also found to widen the perovskite bandgap^34^. Studies found that the blue-shifted emission of Sn-involved Pb-based perovskites is solely determined by Pb–Br octahedral deformation instead of guest Sn-Br groups under a small amount of Sn incorporation, where the host lattice contraction is the decisive factor for bandgap enlargement. Strictly, most replacements of Pb^2+^ with metal ions are not substitution but doping due to the trace amount of addition. Excess B-site metal doping will induce harmful strains or foreign energy transitions to impede the efficiency and color purity of blue emissions.

Mn-doping is a common strategy to enhance the PLQY of perovskite NCs by defect passivation and is verified to improve the stability of green perovskite emitters^41,42^. However, unfortunately, excessive doping of Mn^2+^ introduces an independent transition for the exciton-to-dopant energy transfer and resultant multi/mixed-emissions (Fig. 2e)^43^, as evidenced by an orange-red color of Mn-doped CsPbCl_3_. Therefore, a crucial balance should be concerned in doping contents to make good emissive gains and no Mn-induced decay path in host perovskites. By carefully controlling light doping of 0.19% Mn^2+^, a significantly enhanced PLQY is achieved in CsMn_*y*_Pb_1-*y*_Br_*x*_Cl_3-*x*_ with the emission center at 468 nm^35^. If further increasing the Mn^2+^ dopant, the emission turns purple-red due to emerging Mn transitions at 600 nm. In addition, Y^3+^ doping is found to enhance the blue emission of CsPbBr_2.4_Cl_0.6_ and slightly increase the bandgap without new band incorporation. Interestingly, a gradient distribution of Y^3+^ was identified in perovskite grains from inner to surface (Fig. 2f)^44^, which could be explained by the increased chemical pressure of Y^3+^ species during crystal growth or the doping limitation of Y^3+^ into perovskite lattices. Most of the introduced YCl_3_ accumulated at the grain boundaries and spontaneously formed a type-I band alignment because of the wider bandgap of surrounding YCl_3_ than that of inner grains, finally confining the recombination of exciton in bulk and minimizing interfacial trap-induced losses. At present, B-site substitution still faces doping difficulties according to the structural tolerance, limiting the blue-shifting extent of emissions. To achieve a pure-blue color for display applications, X-site substitution is essential.

### A-site substitution

Despite A-site cations not directly participating in the band edge, their size would affect the deformation of surrounding corner-sharing B-X octahedral scaffolds and the bandgap. Tilt, distortion, and contraction of B-X octahedrons generally are present under the changed chemical pressure by A-site substitution, and many works have concluded an empirical law that in 3D perovskites, smaller A-site cations result in wider bandgaps^45,46^. Among the common A-site cations, Cs^+^ is the smallest, and thus perovskite blue emitters are generally based on CsPbX_3_. Further replacing Cs^+^ with smaller cations, such as Rb^+^, is confirmed to tune the PL emission towards shorter wavelengths^47,48^. Meanwhile, Rb^+^ could regulate crystallization dynamics to reduce the domain size with less film roughness. An abnormal example is that a large organic cation, i.e., CH_3_CH_2_NH_2_^+^ (EA^+^), is successfully employed to achieve a blue-shifted emission by A-site substitution as well (Fig. 2g)^49^. First-principle calculation of the EA-involved CsPbBr_3_ showed the distinct downtrend of CBM and VBM in the energy diagram, where the VBM has a more obvious variation. This phenomenon could be attributed to the uneven expansion of the Pb–Br octahedral lattice because of the anisotropy of EA cations. In deformed octahedrons, the longest Pb–Br orbital by weak coupling determines the CBM, and the shortest counterpart dominants the VBM. Therefore, precisely designing a rational lattice structure by substitution, doping, or strain-induced deformation, is fundamental to enlarging perovskite bandgaps for blue emission.

## Dimensional control

Further increasing the size of A-site cations results in low-dimensional phases by breaking infinite corner-sharing 3D octahedral scaffolds. According to the selected cation, there will be quasi-2D (*n* > 1, *n* denotes the number of B-X octahedron layers between large intercalating cations), 2D (*n* = 1), 1D, and even 0D perovskites with high exciton binding energies, which are desirable emitting candidates for LED applications. However, the large cations, consisting of long-chain organic ammonium, are unfavorable to carrier transportation and recombination in devices because of their insulating nature, which is similar to the surface ligand existing in quantum-dot devices. For high-performance purpose on devices, quasi-2D perovskites, with the advantages of adjustable structure of large cations, are suitable to serve as high-quality emitters with tunable bandgaps for blue PeLED devices.

**Fig. 3:**
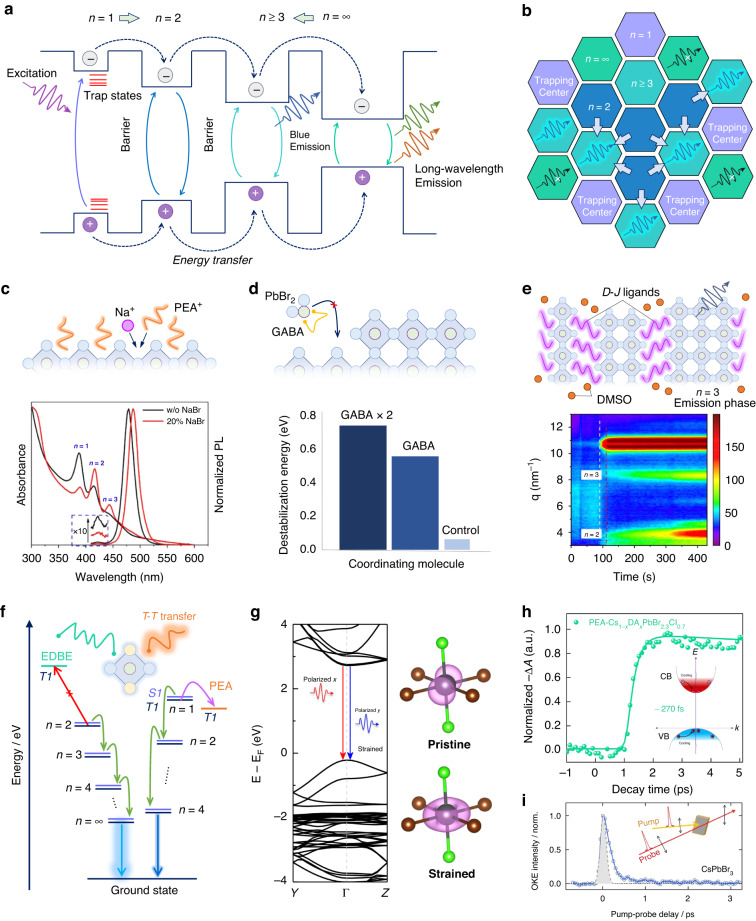
Dimensional control. **a** Charge behaviors in multiple quantum wells of quasi-2D perovskites. **b** Desired energy landscape and domain distributions of the quasi-2D perovskite film. **c** The Na^+^-regulated growth of perovskites for the elimination of the *n* = 1 phase, and the absorption and PL spectra of the quasi-2D perovskites with or without (w/o) 20% NaBr addition. **d** The GABA-induced delayed growth of high-*n* phases by chelating PbBr_2_ precursor, and the destabilization energies of the PbBr_2_ compounds coordinated with control (PEA), GABA, and two GABA. **e** Vapor-assisted phase regulation of 2D D-J perovskite films, and in situ grazing incidence wide-angle X-ray scattering of the evolution of quasi-2D perovskites in DMSO atmosphere. **f** Proposed energy transfer processes of quasi-2D perovskites. *S* and *T* denote singlet and triplet levels, respectively. **g** Simulated band structure of strained CsPbBr_2_Cl, and partial charge densities for the CB-edge state at Γ point of the pristine and strained Pb–Br/Cl octahedron. **h** Ground-state bleaching dynamics probed at the band edge of the DA-involved strained quasi-2D film. Inset shows the hot-carrier cooling process. **i** Time-resolved optical Kerr effect response of CsPbBr_3_. Inset shows the pump-probe experimental setup. **c** is reprinted from ref. ^59^ with permission from the American Chemical Society. **e** is reprinted from ref. ^65^ with permission from the American Chemical Society

As the classical energy diagram of quasi-2D perovskites in Fig. 3a, the energy cascade is a representative carrier behavior in this multi-phase system^50,51^. Free carriers or excitons would transfer from wide to narrowband at an ultrafast picosecond level by inter-band tunneling across the large-cation barrier and emitting low-energy photons, improving radiative efficiencies. In a conventional quasi-2D film, various perovskite phases, such as *n* = 1, 2, 3, are spontaneously formed together during film growth because of their close formation energies. With the descent of the *n* value, the bandgap of these quasi-2D phases gradually opens due to enhanced dielectric confinement effects, satisfying the blueshift of PL emission in this system. However, high-*n* phases, especially the 3D phase (*n* = ∞) with narrower bandgaps, emit low-energy photons by energy cascade, which is undesirable to the blue emission from the middle-*n* phases and should avoid. Moreover, the *n* = 1 phase, which is the most thermodynamically stable phase among this multi-phase system^52^, owns more trap states and strong exciton-phonon coupling^53,54^, resulting in severe nonradiative recombination and retarded exciton transfer. Therefore, to achieve a well-performed quasi-2D perovskite blue emitter, both *n* = 1 and high-*n* phases should be inhibited during the film formation, just leaving *n* ≥ 2 phases with suitable energy landscape and domain distribution for energy cascade and efficient blue emission (Fig. 3b).

### Phase distribution

Many efforts are devoted to modulating the phase distribution of the quasi-2D perovskite emitter. The mainstream strategy to destabilize the *n* = 1 phase is to increase its formation energy by spacer mixing, solubility control, or packaging arrangement^53,55–58^. Variety of A-site spacer combinations, generally a phenyl-ammonium cation combined with a long-chain ammonium cation, have been demonstrated to inhibit the formation of *n* = 1 phase and achieve pure and bright blue emission of quasi-2D perovskites. Besides, the inorganic modulator, such as Na^+^, is proven to increase the *n* value of quasi-2D phases by regulating crystal growth dynamics (Fig. 3c)^59^. The introduced Na^+^ competitively occupies A-site with phenylethylammonium cation (PEA^+^) during crystallization, and the resultant Na-substituted *n* = 1 phase maintains a shorter time because of its larger formation energy than that of PEA-based counterparts. Therefore, the proportion of high-*n* phases, especially *n* = 2 and 3 phases, is correspondingly increased as confirmed by optical characterizations, where the red-shifted PL emission could attribute to the increment of Br content or enlarged domain size upon NaBr introduction. Benefiting from rearranged phases by Na^+^, the exciton transfer is accelerated from *n* = 2 to high-*n* phases, resulting in bright and stable sky-blue emission at 488 nm.

Suppressing high-*n* phases is to avoid redshift of the emission profile. For example, blue quasi-2D perovskite emitters tend to emit green light due to the existence of high-*n* phases, even though the concentration of high-*n* phases is low^60^. Inhibiting the overgrowth of thick perovskite layers in the precursor is critical to rational phase distribution and pure emission. A chelating agent, *γ*-aminobutyric acid (GABA), is employed to coordinate with PbBr_2_ and improve the solubility of this compound in the precursor (Fig. 3d)^61^. Tenfold destabilization energy is obtained from the PbBr_2_ compounds coordinated by bidentate GABA (0.51 eV) compared with the unidentate control group (0.05 eV), and can be further increased by utilizing two GABA for dual coordination. The increased destabilization energies are responsible for the delayed growth of Pb–Br octahedral lattices at the most active step edges, finally leaving low-*n*-dominated phases for purer blue emission.

In addition, the bidentate ammonium is popular to serve as the spacer to the formation of quasi-2D Dion–Jacobson (D-J) phases with narrower Van der Waals barriers^62,63^, which facilitates exciton tunneling and transportation. However, the formation energies of D-J phase are significantly increased with the increment of *n*^64^, impeding middle-*n* D-J phases during film growth and thus creating an exciton tunneling gap. To overcome this, a dimethyl sulfoxide (DMSO) vapor treatment successfully optimizes crystal growth and modulates the phase distribution of D-J perovskite films (Fig. 3e)^65^. In situ crystallization kinetic tracing of the DMSO-treated film confirms the suppression of *n* = 1 and 2 phases and the incremental *n* = 3 phase, which derives from alleviative spacer aggregation and accelerated spacer migration in perovskite scaffolds with the help of DMSO molecules. Based on rearranged phase distribution and well-matched band alignment, resultant blue PeLEDs achieved remarkable external quantum efficiencies (EQEs) of 13.7% and 15.5% with emission peaks at 489 and 494 nm, respectively.

### Energy landscape

In comparison to 3D counterparts, quasi-2D perovskites present more a complex energy landscape, for example, multiple excited-state transitions, hot-carrier cooling, trap-assisted annihilation, triplet energy transfer, exciton inter-band tunneling, exciton–exciton annihilation and so on^30,66–68^. Improving the ratio of radiative recombination and avoiding nonradiative losses are critical to managing the energy landscape of quasi-2D perovskite emitters for efficient light emission. Many works focused on suppressing trap-assisted recombination paths by passivating bulk and interfacial defects^69,70^, weakening electron–phonon coupling or eliminating defective *n* = 1 phase. In addition, the spacers in quasi-2D perovskites could probably induce triplet energy losses in Pb–Br perovskites by the small energy gap between singlet and triplet states^66,71,72^, approximately equal to the thermal activation energy at room temperature. A triplet–triplet Dexter transfer occurs when the triplet level of spacers is lower than that of the inorganic octahedron, as shown in Fig. 3f, where exciton transfers from radiative recombination center to spacer, leading to nonradiative losses. Therefore, the employed spacer is expected to have a higher triplet level to avoid this competitive energy consumption path. A dual-functional ammonium, 2,2-(ethylenedioxy)bis(ethylammonium) (EDBE), with a high triplet level of 5.23 eV, effectively closes the triplet transfer channel and substantially passivates trap states to mitigate these energy losses of quasi-2D blue emitters^66^. A thermally activated delayed fluorescence is discovered in the EDBE-based film, experimentally confirmed by the reverse intersystem crossing from triplets to singlets without Dexter transfer. In brief, spacers are not only a barrier to exciton confinement in quasi-2D perovskite layers but a modulator to form a rational energy landscape for exciton transfer and recombination.

The landscape of excited-state transitions determines emission efficiencies and depends on the transition dipole moment (TDM), which in turn determined by the lattice structural dynamics^73^. According to the simulated electronic structure of blue emitter CsPbBr_2_Cl, the evaluated TDM intensities between VB and three CB-edge states indicate three different transition bands with different polarization modes named polarized *x*, polarized *y*, and polarized *z*^74^. Only *x*-polarized transition is allowed for pristine perovskites whereas the other two transitions are forbidden. When a 4% tensile strain is applied to the perovskite lattice along the *x* direction, the bandgap is slightly enlarged, and especially, *x*- and *y*-polarized transition modes are merged into a quasi-degenerate state around the Γ point at the CB edge (Fig. 3g)^74^. Meanwhile, the strained octahedron performs overlapped charge densities at the *xy* plane. This double-polarized mode in the strained system would remarkably enhance the TDM intensity, and thus the probability of radiative transitions. Diethylammonium (DA) cation was first attempted to modulate the lattice structure and fine-tune the lattice strain of the PEA-involved quasi-2D CsPbBr_2.3_Cl_0.7_, effectively opening radiative transition channels and significantly improving their PLQYs as predicted. Ultrafast spectroscopies in-depth studied on carrier dynamics of this strained quasi-2D film and found an accelerated hot-carrier cooling process with a time of 270 fs (Fig. 3h). Long-lived hot carriers are the unique feature of lead-halide perovskites because the existence of delocalized large polarons, formed by electron–phonon or vibronic coupling between charge carriers and perovskite ionized lattices, could screen and delay the energy scattering of hot carriers^75^. Organic-involved perovskites are more likely to produce large polarons because of the anisotropic reorientation motion of A-site cations. Although the time-resolved optical Kerr effect responses do not reveal the liquid-like re-orientational dynamics in inorganic CsPbBr_3_ (Fig. 3i)^76^, the deformation of soft Pb–Br octahedron scaffolds is confirmed to couple with charge carriers for the formation of polarons^77^. Therefore, hot-carrier cooling is a common behavior in the CsPbBr_3_ host for blue emission and is considered to have a competition with charge trapping from higher energy levels during the energy cascade. The fast-cooling process will be good at the electron–hole radiative recombination in quasi-2D perovskites and is a good idea for designing perfect energy landscapes.

## Size confinement

**Fig. 4:**
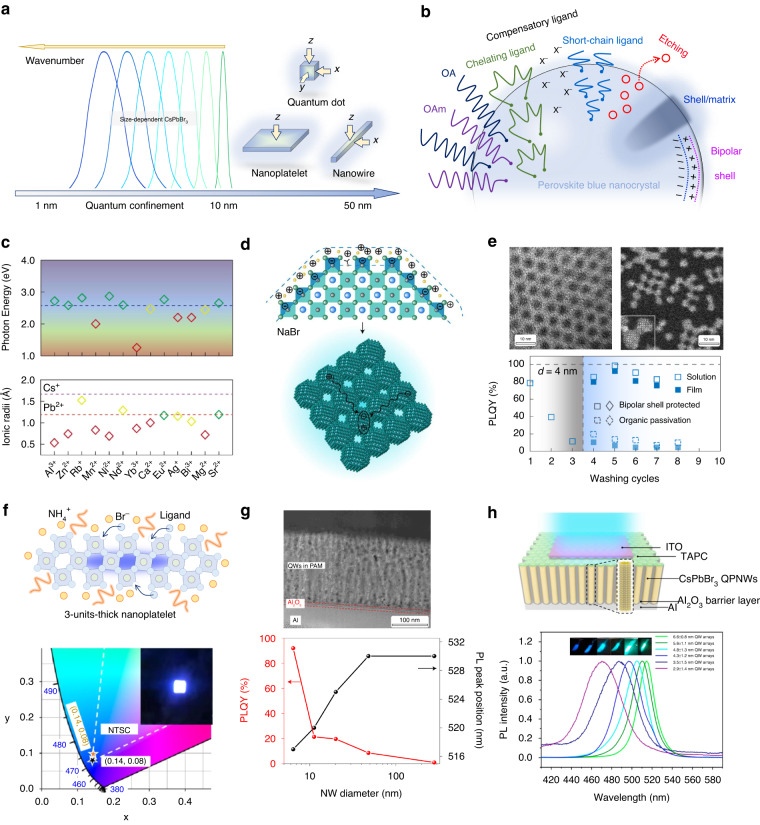
Size confinement. **a** Size-dependent PL spectra of CsPbBr_3_ and three conventional forms of perovskite blue NCs. **b** Various surface optimization strategies of perovskite blue NCs. **c** The emitting photon energy of doped QD-in-matrix emitters and the ionic radii of candidate dopants. **d** Schematic of the resurfaced perovskite QD with a bipolar shell and the solid formed with resurfaced. **e** Transmission electron microscopy (TEM) image of purified perovskite QDs with organic ligands, and low-dose scanning TEM Z-contrast image of resurfaced perovskite QDs (top). Inset shows two adjacent QDs. The PLQYs of QD colloidal solution and solid films made from organic-passivated QDs and bipolar-shell-stabilized QDs (bottom). The gray area represents washing iterations without ligand exchange process and the blue area represent iterations using bipolar exchanges or organic ligand exchanges, respectively. **f** A 3-unit-thick perovskite NPL enabled by NH_4_Br modulation, and The Commission Internationale de l’Echlaiage (CIE) color coordinate of the blue PeLED devices. Inset shows the photograph of the NPL-based device with an active size of 4 mm^2^ at an applied current density of 52 mA cm^−2^. **g** Cross-sectional TEM image of the QWs in PAM templates, and PLQY and PL peak position of NW arrays with different porous diameters. **h** PAM-assisted PeLED device, and PL spectrum of CsPbBr_3_ QW arrays grown inside PAM template with different diameters. QPNW: quantum-confined perovskite nanowires. **c** is reprinted from ref. ^84^ with permission from the American Chemical Society. **d**, **e** are reprinted from ref. ^85^ with permission from Springer Nature. **f** is reprinted from ref. ^80^ with permission from the American Chemical Society. **g** is reprinted from ref. ^95^ with permission from Springer Nature. **h** is reprinted from ref. ^96^ with permission from Springer Nature

Besides compositional and dimensional control, size confinement is another strategy for blue emission. There will be a quantum-confinement effect when perovskite domains reduce below their exciton Bohr radius, which is generally less than 10 nm, contributing to the enlargement of bandgap and blueshift of PL emission (Fig. 4a)^78^. Without unreasonable energy landscape and phase segregation in mixed-ion systems, perovskite nanoscale domains, especially the perovskite NCs with the size less than Bohr radius, take advantage of significant confinement effects to achieve blue emissions based on the single-perovskite component (i.e., CsPbBr_3_). In specific, 0D QDs, 1D NWs, and 2D NPLs with quantum confinement are widely studied as perovskite blue emitters and have made great research progress.

Regardless of NCs’ morphologies, a rational synthesis of high-PLQY NCs with good confinement is the prerequisite to high-efficiency blue PeLEDs. Carefully controlling synthesis parameters is facile to adjust the size of resultant NCs, realizing suitable bandgaps and desired emission wavelengths by the confinement extent^78^. Despite blue perovskite NCs having achieved pure and near-unity PLQYs at present, the performance of NC-integrated PeLED devices is still inferior and highly relevant to surface ligands. In the conventional NC synthesis process, i.e., hot-injection process or ligand-assisted reprecipitation, oleylamine (OAm) and oleic acid (OA) are employed as ligands for confined crystal growth and stable colloidal solution (Fig. 4b)^79^. While their insulating hydrocarbon chains anchoring at the NC surface results in poor conductivity and significantly hinders charge injection in working devices. Ligand exchange is a usual preprocessing before device integration to optimize the surface state of NCs, exchanging long-chain OAm and OA with short-chain ligands, chelating ligands, or compensatory ligands^80–82^. Surface optimization could also be realized by imperfection etching, shell/matrix protection, and bipolar-shell design^83–85^, to finally reduce surface defects, enhance confinement effects, decrease charge transport barriers, and improve the stability of NCs in PeLEDs. In short, a surface is a critical place that deserves more effort to these NCs with high specific surface area.

### Quantum dots

As the representative object of size confinement, perovskite QDs have been widely studied for efficient blue PeLEDs because of their facile fabrication protocol and high repeatability. QDs are chosen as the basis to clarify the effect of ligand exchange, metal doping, defect passivation, and other optimizations, where some of which are inherited from the experiences on bulky films. QD-in-matrix strategy has been demonstrated to enable superior surface passivation, leading to efficient and stable QLED devices^84,86,87^. The epitaxial matrix is responsible for the protection and passivation of embedded QDs and also charge confinement by the wide-bandgap matrix shell, which seems a good alternative for organic ligands. Small lattice mismatch and type-I band alignment are essential to effective QD-in-matrix, and it will be stricter to design a suitable matrix for the blue perovskite QD with a wider bandgap. To construct a desired QD-in-matrix for strongly confined CsPbBr_3_ QDs with sky-blue emissions, a Sr-involved perovskite matrix with a wide bandgap and matched lattice was screened out (Fig. 4c)^84^. Because X-site substitution tends to cause halide segregation and lattice mismatch, B-site metal doping is more suitable for the bandgap opening of the surrounding matrix. Potential doping candidates are investigated according to their ionic radii and the PL photon energy of the matrix, and Sr^2+^ was selected as the most suitable dopant for the CsPbBr_3_ host matrix. To avoid the intrinsic hygroscopicity of Sr^2+^, a passivator is introduced to ensure the reliability of the perovskite matrix and the whole QD-in-matrix system. The resultant sky-blue PeLEDs showed a champion EQE of 13.8% and a brightness exceeding 6000 cd m^−2^, plus enhanced spectra stability.

In perovskite QDs, the non-covalent binding between ligands and QDs leads to a dynamic ligand absorption-desorption phenomenon^88^, which destabilizes the QD surface and weakens radiative efficiencies. Moreover, organic ligands generally impede charge transport in devices mentioned above. Therefore, fewer organic ligands are a development trend for QD PeLED devices. For this purpose, a bipolar shell with the help of atomic ligands is proposed to be an outstanding stabilizer to perovskite QDs (Fig. 4d)^85^. By monitoring electrokinetic potential, three-step post-treatment on as-synthesized QDs, including purification, Br^-^ compensation, and Na^+^ substitution, is verified to form a bipolar shell on perovskite QDs. The resurfaced QDs are predicted to assemble into solids with a short inter-QD distance that enables efficient charge transport. And the reduced interparticle spacing from >2 nm to atomic scale after resurfacing is confirmed by the high-resolution electron microscopy (Fig. 4e). After resurfacing process, conventional V_X_ defects are thoroughly passivated or compensated by Br^-^ inner shell, and the corresponding PLQYs exhibit a noticeable improvement to nearly 100%. Furthermore, regardless of the colloidal QD solution or the QD film, the Na^+^-enriched outer shell effectively suppresses ligand-ion pair dynamic equilibrium during several iterations of purification, contributing to enhanced PLQY stability. The resurfaced 4-nm CsPbBr_3_ QDs with superior size confinement realized a blue emission at 480 nm and finally achieved a champion PeLED device with the best EQE of 12.3%.

### Nanoplatelets

Perovskite 2D NPLs with two-dimensional space confinement, taking advantage of narrow PL peaks, anisotropic luminescence, and thickness-tunable emission, have shown huge potential for high-outcoupling PeLEDs^89,90^. Highly oriented perovskite NPL emitters with a high ratio of horizontal transition dipole moments have been demonstrated to boost the photon outcoupling of green PeLED devices^91^. In particular, because of the tunable thickness, the emission wavelength of CsPbBr_3_ NPLs could be tuned to the pure-blue region by vertical space confinement. Therefore, effectively controlling the thickness and surface state of NPLs is highly desirable for the fabrication of pure-blue PeLED devices^92^. By employing NH_4_Br surface stabilizer, ultrathin NPLs with a 3-unit-cell thickness were synthesized (Fig. 4f)^80^. The strong affinity between NH_4_^+^ and [PbBr_6_]^4-^ stabilizes the growth of NPLs and promotes the formation of a Br-rich passivated surface. The resultant CsPbBr_3_ NPLs with short ligands possess uniform thickness distribution and realize a pure-blue emission at ~460 nm with the assistance of the ultrathin vertical confinement. Similar to the bipolar shell in QDs, small cations plus halide anions (i.e., Br^-^) are verified as an efficient ligand combination for highly efficient and stable perovskite NCs. With the help of the post-treatment of short conjugation ligands, an attractive pure-blue EL efficiency of 2% was enabled by the ultrathin CsPbBr_3_ NPL emitter, where the emission peak corresponds to CIE color coordinate of (0.14, 0.10).

### Nanowires

Perovskite 1D NWs or nanorods hold their superiorities in highly directional charge injection, strong one-dimensional size confinement, and orientational propagation of light emission. Although various preparation methods are developed for the synthesis of 1D NWs and nanorods^93,94^, integrating them into LED devices is still challenging because of various technical difficulties, especially the anisotropic arrangement after deposition. Building a vertically arranged 1D NW array with a preferred light emission direction is essential to unlocking the applications of perovskite NWs in LED devices. To achieve a uniform large-scale NW array, a porous alumina membrane (PAM) template is employed to assist the growth of perovskite NWs and control their size confinement by adjusting the pore diameter^95,96^. Recently, a high-crystalline perovskite quantum-wire (QW) array was fabricated by filling perovskites into PAMs with the a pore diameter of below 10 nm (Fig. 4g)^95^. Benefiting from the quantum-scale confinement, the PLQY of perovskite QW arrays remarkably reaches more than 90% and the bandgap opens. Moreover, the hydrophobic PAM template could protect perovskite NWs against external moisture and alleviate ion migration to a great extent. By employing the PAM-assisted CsPbBr_3_ QW emitter combined with dedicated device configuration^96^, blue PeLED devices were enabled with cyan (492 nm), sky-blue (481 nm), and pure-blue (467 nm) emission colors by the tunable pore size of PAMs (Fig. 4h). PAM templates are also promising towards future micro-PeLED applications because the naturally separated microscale pore units result in many isolated LED devices that can be addressed independently^97^.

## Stability progress

The stability of perovskite blue emitters is highly concerned in the community. Unlike the perovskite active layer employed in photovoltaics, the counterparts for LED applications generally possess a thinner thickness and suffer from a higher bias voltage in operation. The intrinsic weaknesses of perovskites, for example, ion migration, organic volatilization, etc., would be aggravated, and thus the device stabilities of PeLEDs are significantly worse than their photovoltaic counterparts at present. In short, stability issues hinder the commercial progress of PeLED devices and should be in-depth studied and resolved.

**Fig. 5:**
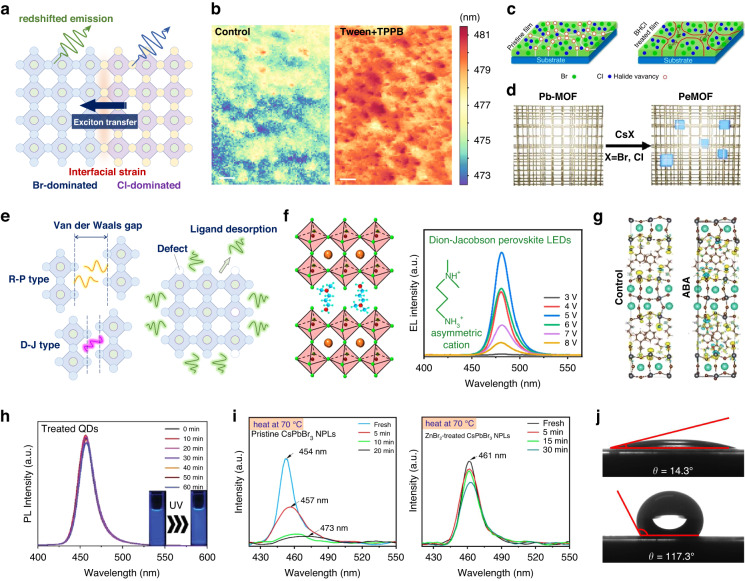
Stability progress. **a** Phase segregation of mixed-halide perovskites. **b** The distribution of emission peaks extracted from the cathodoluminescence mapping of control and Tween+TPPB perovskite films. **c** Schematic of the interfacial passivation of the mixed-halide perovskite film by BHCl. **d** Schematic of the growth of CsPbX_3_ NCs in MOF matrix. PeMOF perovskite-MOF. **e** The Van der Waals gap in quasi-2D perovskites and surface imperfections in perovskite NCs. **f** The lattice structure of DPDA-based D-J quasi-2D perovskites, and the EL peak evolution of the DPDA-based PeLED device under different applied bias. Inset shows the molecular structure of DPDA. **g** The deformation charge density distributions of the control and ABA-treated perovskite films based on the density functional theory simulation. **h** PL evolution of the treated QDs under ultraviolet irradiation for different times. Inset shows photographs of treated QDs after UV irradiation for different times. **i** Temporal evolution of PL intensity for pristine and the ZnBr_2_-treated CsPbBr_3_ NPLs dispersion under 70 °C heating. **j** Water contact angle measurement of PAM with and without hydrophobic surface treatment. **g** is reprinted from ref. ^114^ with permission from Wiley. **h** is reprinted from ref. ^83^ with permission from Wiley. **i** is reprinted from ref. ^115^ with permission from the American Chemical Society. **j** is reprinted from ref. ^95^ with permission from Springer Nature

Among three-color perovskite emitters and their PeLED devices, blue is the most unstable^98^. Three classical categories of perovskite blue emitters face distinct stability issues. The halide-mixed perovskites are prone to phase segregation upon external stimuli including thermal, photo, moisture, etc. (Fig. 5a). Especially, low diffusion activation energies of Cl^−^ would accelerate the accumulation of Cl-dominated perovskite domains, which was verified by the in situ PL spectra that presents multiple emission peaks^99^. As the migration process goes on, an obvious red-shifting of emissions was observed due to the exciton transfer from wide-bandgap Cl domains to narrow-bandgap Br domains. Moreover, the interface between Cl and Br domains would be an unstable region for perovskite decomposition because of mismatched lattice parameters. Employing single-halide perovskites, i.e., low-dimensional perovskites and perovskite NCs, could effectively avoid phase segregation. While the introduced spacer/ligand in these two forms is the key to unstable issues of perovskite blue emitters (Fig. 5e). Spacer-induced Van der Waals gap means a weak interaction between perovskite lattices^100^, which could deteriorate the stability of the quasi-2D perovskite structure. Meanwhile, the ligands on perovskite NCs could easily desorb from the surface^85^, resulting in rich surface defects and even NC fusion, which are harmful to spectral stability. Substantial progress has been made recently in the improvement of material stability and in providing meaningful guidance to stable blue PeLED devices. In the following, we would like to discuss the representative works focusing on these types of blue perovskites.

### Mixed-halide blue emitters

In general, halide migration is deemed to derive from the rich defects in perovskites that launch halide ions moving towards somewhere and accumulating in domains. Therefore, many efforts focused on the passivation of bulk or interfacial defects of as-formed perovskite films to inhibit phase segregation^101^. However, unevenly distributed halides in the original perovskite film, probably caused by the distinct nucleation and crystallization kinetics in precursor, are always neglected, which could be one of the reasons for subsequent halide migration. To solve this issue, a polymer polyoxyethylene sorbitan monolaurate (Tween) combined with a cationic surfactant tetraphenylphosphonium bromide (TPPB) was employed to synergistically homogenize the halide distribution (Fig. 5b)^102^. It was found that Br and Cl species are not fully mixed in the pristine precursor, and their separate crystallization results in an inhomogeneous phase distribution. The introduced TPPB serves as the intermediary to promote halide exchange from their respective domains, and Tween weakly binds with Cs^+^ to release more Br^-^ to participate in halide exchange in the precursor. Cathodoluminescence mapping confirms the homogenized emission from the optimized perovskite domains, and the resultant blue PeLED device performs stable EL spectra at 482 nm. Furthermore, multi-source vacuum deposition and vapor-assisted reconstruction are also efficient solutions to halide homogenization discussed in “X-site substitution”.

For the as-formed perovskite film, interfaces and grain boundaries are the defect reservoir^24,103^, especially V_X_, of polycrystalline films and are responsible for halide migration upon external stimuli. The most popular passivator towards interfacial defects is the ammonium-halide salts that are widely studied to clarify their abilities to not only bind with uncoordinated Pb but compensate for halide vacancies^104–106^. Therefore, a chloride salt benzamidine hydrochloride (BHCl) was employed to passivate interfacial halide vacancies and suppress ion-migration channels (Fig. 5c)^107^. The added BHCl can also regulate the film growth and result in a preferential crystal orientation with reduced defect densities. Encouraged by efficient passivation, the BHCl-treated film presents better photostability and realizes spectral-stable devices with higher luminance and efficiency. While these passivation effects are still limited because the introduced passivators cannot fully restrict halide migration in a nanoscale domain. Recently, a creative strategy applied metal-organic frameworks containing Pb nodes (Pb-MOF) to confine the in situ growth of perovskite NCs embedded into the MOF matrix (Fig. 5d)^108^. By simply introducing CsX (X = Br, Cl) precursor into the as-formed Pb-MOF film, ~20-nm CsPbBr_3_ and CsPbCl_3_ NCs, instead of the alloyed CsPb(Br/Cl)_3_ NCs, grew into the MOF scaffold, as revealed by TEM measurements. Interestingly, mixed perovskite NCs in MOFs do not exhibit two emission peaks but only one according to the ratio of introduced CsBr/CsCl. And the related mechanism of this phenomenon should be further explored. Benefiting from the protection of stable MOF scaffolds and intrinsically inhibited halide migration, the hybrid blue emitters sustain much higher laser power irradiation against light-induced ion migration than the CsPbBr_3−*x*_Cl_*x*_ film.

Perovskite light-emitting electrochemical cells (PeLECs), belonging to another electroluminescence (EL) device, are promising to demonstrate stable emissions benefitting from their simplified device configuration and blend-stabilized emitting layers^109,110^. Especially for Cl-involved ion-mixed blue emitters, such as the familiar CsPbBr_3-*x*_Cl_*x*_, the undesired ion migration of perovskites could be effectively restricted, and resultant blue PeLECs exhibited more stable EL spectra^111,112^. Although the development of PeLECs is still in the initial stage, this unique configuration would be an efficient technical path towards stable and efficient perovskite blue EL devices based on 3D mixed-ion perovskite active layers.

### Single-halide blue emitters

Employing single-halide perovskites as blue emitters is the growing trend for efficient and stable devices because of the eliminated phase segregation. However, the substitutes, including quasi-2D perovskites and perovskite NCs, possess rich organic spacers and surface ligands that could be other unstable factors. To narrow the Van der Waals gap in conventional PEA-based quasi-2D perovskites, the D-J phase is a good choice and has been widely demonstrated to show improved stability in optoelectronics. The bidentate ammonium spacer binding with two adjacent perovskite lattices could narrow the gap according to the spacer size and effectively stabilize the whole system through strong interactions. Especially, an asymmetric spacer named *N*,*N*-dimethyl-1,3-propanediamine (DPDA), was proved to efficiently keep the phase stability of D-J perovskites better than that of their symmetric counterpart (Fig. 5f)^113^. The dipole moment of the DPDA spacer is proposed to interact with V_X_ to the dispersion of local charges, relieving octahedral distortion and increasing the ion-migration barrier. It is verified by a small lattice expansion of the asymmetric DPDA-based perovskite film, further indicating a rigid perovskite framework could strengthen the structure stability of the quasi-2D blue emitter. Under increased bias, the DPDA-based PeLED device showed superior spectral stability. Except for the bidentate ammonium spacer, the ammonium with a functional end group also satisfies the reduction of the Van der Waals gap in quasi-2D perovskites. A bifunctional ammonium 4-(2-aminoethyl)benzoic acid (ABA) was chosen as the spacer that could bind with uncoordinated Pb by O terminal groups, to narrow the gap between two perovskite layers (Fig. 5g)^114^. Simulations confirmed strong interactions between the ABA spacer and perovskite lattice, and the operational half-lifetime of the resultant PeLED device is 2.5-fold longer than that of the original device.

The immense surface-to-volume ratio of perovskite NCs means the surface quality is of great importance to material stability. Great attention has been focused on the optimization of NC surface lattices and anchored ligands. Pre-etching by HBr acid is going to remove surface imperfect octahedral lattices of small-sized CsPbBr_3_ QDs^83^. The added HBr in the precursor owns the backward reaction with conventional OA and OAm ligands and promotes the ligand protonation and desorption. The surface V_Br_ and imperfect lattice of poorly crystalline QDs would be etched, and the Br-rich surrounding of residual high-quality QDs is responsible for the V_X_-free surface. With the aid of the short-ligand exchange for surface stabilization, the etched QDs showed superior ultraviolet resistivity under 60-min irradiation (Fig. 5h). The surface imperfections are found in perovskite NPLs as well. ZnBr_2_ was employed to post-treat as-formed CsPbBr_3_ NPLs to reduce the possibility of coalescence and degradation and improve the colloidal stability against storage, polar solution, light, and heating^115^. Stressing under 70 °C (Fig. 5i), pristine NPLs underwent coalescence within just 10 min according to the redshift evolution of the PL emission, while the ZnBr_2_-treated NPLs still showed good color stability exceeding 30 min. Note that the Zn^2+^, anchored around the NPL surface by interactions with Br^-^, is responsible for the inhibition of Br migration. Improving the stability of NCs by template protection is a good idea. As discussed above^95^, the surface-treated PAM template performs a good hydrophobicity (Fig. 5j), preventing perovskite NWs from external moisture. The resultant perovskite NW film possesses excellent water-repellent properties and exhibits good spectral stability (~1% efficiency drop) stored in ambient air for five days (at 23 °C and ~45–55% relative humidity).

## Future perspectives

Three mainstream trends of perovskite blue emitters are systematically reviewed and comprehensively discussed. We conclude that the inferior performance of blue PeLEDs than their red or green counterparts could derive from three primary causes including rich imperfections, ion migration, and unmatched alignment. The wider bandgap of blue emitters means a broader energy region for the presence of trap states, especially for Cl-involved perovskites that could introduce rich deep-level states^25,26^, resulting in serious nonradiative recombination. And in practice, wide-bandgap perovskites still face poor film qualities due to the complex and uncontrollable crystallization and growth processes^30^. For the commonly employed perovskite polycrystalline film, most grain boundaries and interfaces serve as defect reservoirs, which further aggravate the trap-induced recombination and even film degradation. Ion migration is negligible but a critical issue for mixed-ion blue perovskites^28,29^. The resultant phase segregation would become another source of imperfections and non-ideal recombination regions in blue perovskite emitters. Furthermore, in the device configuration, it is difficult to find a suitable hole transport layer that could well match with deep VBM of blue perovskite emitters^116–118^. Therefore, interfacial energy losses are also responsible for poor blue device performance.

With the maturity of experimental techniques and an in-depth understanding of the perovskite material, the technical roadmap for high-quality blue emitters and the fabrication of state-of-the-art PeLED devices become clearer. Among these three optimization strategies, dimensional control belongs to a kind of compositional engineering in a sense because the essence of dimensional control is A-site substitution from small to large cations. A comprehensive compositional design including A-, B-, and X-site engineering towards perovskites with desired emission peaks, stable lattices, pure phases, and rational energy landscapes, seems to be the ideal strategy for the achievement of perfect blue emitters. While in practice, the ideal composition could lead to unexpected complex crystallization dynamics and resultant films with heterogeneous phases. For example, mixed-halide perovskite blue emitters are outdated and rarely studied at present because of unavoidable phase segregation and abundant Cl-related deep-level trap states, despite their ideal composition with rational bandgaps. By contrast, single-halide perovskites, i.e., pure-Br low-dimensional or nanosized perovskites, with the space/quantum-confinement effect are the most popular candidates and show great potential for the development of highly efficient and ultra-stable emitters and devices.

**Fig. 6:**
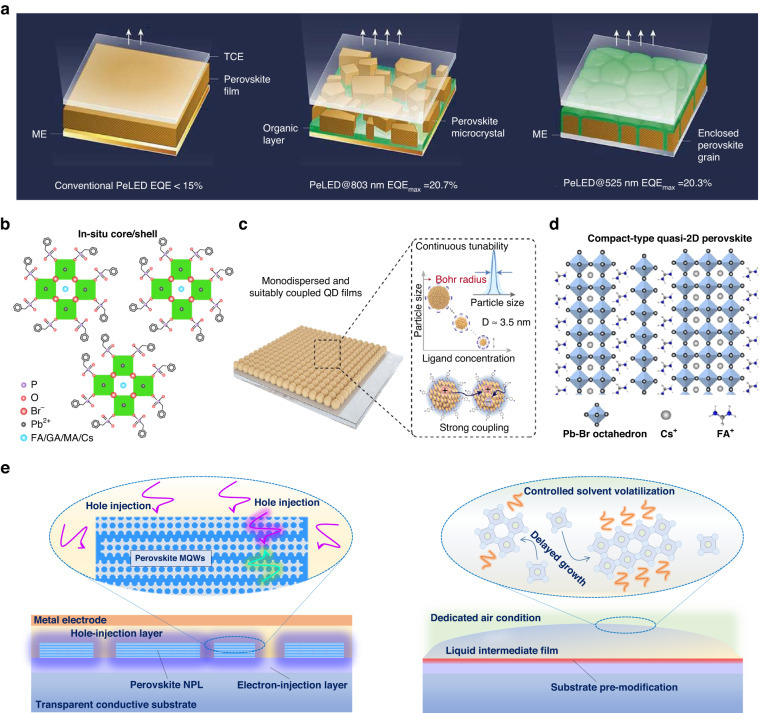
Future perspectives. **a** Schematic of three types of PeLEDs with different architectures and perovskite emitters. ME metal electrode, TCE transparent conductive electrode. **b** In situ formed core/shell perovskite NCs by the introduced benzylphosphonic acid. **c** Monodispersed perovskite QD films achieved by the synthesis-on-substrate method, and the related quantum-confinement and strong-coupling effects. **d** The lattice structure of compact-type quasi-2D perovskites. **e** Proposed ideal perovskite blue NPLs in the dedicated device, and the controlled growth of perovskite NPLs from liquid intermediates on the pre-modified substrate in mild conditions. Note that the ligands are expected to locate at the interfaces between two charge-injection layers in the device. **a** is reprinted from ref. ^97^ with permission from Springer Nature. **b** is reprinted from ref. ^7^ with permission from Springer Nature. **c** is reprinted from ref. ^122^ with permission from Springer Nature. **d** is reprinted from ref. ^123^ with permission from the American Chemical Society

Lessons from the reported high-efficiency PeLEDs are meaningful to summarizing take-home messages for predicting the technical roadmap of blue emitters. In 2018, two PeLED breakthroughs were made simultaneously in green and near-infrared emissions^119,120^, realizing device efficiencies exceeding 20% for the first time (Fig. 6a). The near-infrared emitter is the submicrometre-scale isolated FAPbI_3_ crystals (FA: formamidine) by a specialized amino acid regulation during spin-coating^119^. The green CsPbBr_3_@MABr emitter owns a quasi-core/shell microstructure, presenting nanoscale-isolated perovskite emitting domains as well^120^. These isolated domains collectively indicate that space confinement is a critical commonality to the achievement of radiative recombination^121^, boosting the efficiencies of resultant PeLED devices.

Very recently, a record efficiency of 28.9% of the green PeLED device was enabled by in situ formed core/shell perovskite NCs with a size of approximately 10 nm on the substrate without a separate synthesis process (Fig. 6b)^7^. Similar to isolated near-infrared FAPbI_3_ crystals mentioned above, the perovskite core/shell nanostructure was realized by the introduced benzylphosphonic acid “ligand” that could penetrate and intercalate into crystals, splitting a large domain into nanosized crystallites. Note that the size of crystallites is about or even less than 10 nm, which means significant charge confinement in NCs for strengthening radiative recombination. More importantly, almost at the same time, champion blue PeLED devices with the record EQEs of 17.9% at sky-blue 480 nm and 10.3% at pure-blue 465 nm, was achieved based on CsPbBr_3_ QDs with different domain sizes^122^. Interestingly, the domain sizes are less than its Bohr radius of ~3.5 nm and are realized by the direct synthesis-on-substrate from the precursor but not the traditional QD synthesis process (Fig. 6c). A dedicated ligand with a high steric hindrance head group and an electron-attracting end group is designed as the additive to bind at the CsPbBr_3_ surface to regulate the formation of strongly coupled, monodispersed, ultrasmall QDs on substrates. With the increment of the ligand content, the QD size could be tuned from 6.4 nm to 3.5 nm, and the PL emission from green to pure blue. Moreover, this novel methodology is universal to other CsPbX_3_ emitters that could almost cover the whole emission spectrum for display applications.

From recent advances in highly efficient PeLEDs, we conclude the developing trend of the ideal form of high-quality perovskite blue emitters. The size of the active emitting domain gradually decreases from the microscale to the nanoscale, and finally to the quantum scale. Meanwhile, the fabrication process is almost based on the one-step spin-coating with additives that take on the role of ligands for the growth of nanosized domains. Especially, the added ligands generally possess a short chain with the conjugate benzene group, which could minimize charge-injection losses at interfaces. Therefore, we predict that the isolated, single-crystalline, less-ligand, or ligand-free perovskite quantum-scale emitting domains will be the next-generation candidate for high-performance blue PeLED devices. Meanwhile, the one-step fabrication is essential because the direct nucleation and growth of crystals on the substrate could ensure good contact with substrates. To be specific, in situ fabricating 2D NPLs on the substrate will further strengthen the light-outcoupling efficiency. A lateral multiple quantum well structure, inherited from traditional GaN/InGaN LED devices, is expected to be constructed in 2D NPLs by introducing rational ligands and carefully controlling the growth of quasi-2D phases, efficiently confining injected charges in the atom-level space and boosting the radiative efficiency. In addition, formamidine (FA^+^), belonging to a conventional A-site cation of 3D perovskites, is found to form compact-type quasi-2D perovskites with Cs^+^ (Fig. 6d)^123^, which is also a promising quasi-2D candidate that could take into account both charge confinement and transportation. The FA^+^ (2.53 Å) was considered the smallest interlayer “spacer” in Cs (1.67 Å)-dominated perovskites, where the CsPbBr_3_ rapidly crystallizes into inorganic host frameworks during film growth and the larger FA^+^ with two amino groups would bond at the framework surface, preventing further [PbBr_6_]^4−^ stacking. Unlike conventional large 2D spacers, FA^+^ is considered not to affect the isotropy of charge transportation regardless of crystal orientations and leads to an ideal energy cascade in the inorganic perovskite system.

As mentioned above, a shortcoming of blue PeLEDs is inefficient hole injection because of unmatched band alignment. Therefore, from bottom to top, a device configuration comprising transparent conductive substrate/electron-injection layer/perovskite/hole-injection layer/metal electrode would be well-matched with the in situ formed quasi-2D NPL layer. The isolated NPLs on substrates expose more surface area to contact with the top hole-injection layer, improving the hole-injection efficiency to resolve charge imbalance in devices. Balanced charge injection is also beneficial to maintain the stability of the perovskite active layer and operating devices.

Therefore, we propose an ideal perovskite blue emitter integrated into the dedicated device configuration in Fig. 6e. The fabrication process should focus on not only the selection of additives/ligands in the precursor to directly regulate the growth of perovskite NPL crystallites, but the pre-modification of substrates that could induce the optimization of the distribution, size, thickness, and various properties of NPLs. Furthermore, it is more desired to create a mild condition for the delayed growth of single-crystalline NPLs. Therefore, more attention should be paid to the liquid stage between the beginning of spin-coating and thermal annealing, where the room-temperature placement of the perovskite intermediate film is generally overlooked but is crucial to the growth of NPLs. This stage is considered to a self-synthesis process of nanocrystallites which is similar to the solution synthesis of NCs.

In brief, this review systematically concludes recent advances of three mainstreams of the perovskite blue emitter and comprehensively discusses the meaningful stability progress. A future technical roadmap is proposed to guide the fabrication of ideal perovskite blue emitters in a suitable device stack. Blue PeLED devices with pure-blue emission at ~465 nm still lag far behind their green and red counterparts for display applications, and high-quality perovskite blue emitters are believed to be the foremost part that demands more effort on it.
